# Beyond Structure: Defining the Function of the Gut Using Omic Approaches for Rational Design of Personalized Therapeutics

**DOI:** 10.1128/mSystems.00173-17

**Published:** 2018-03-06

**Authors:** Casey M. Theriot

**Affiliations:** a Department of Population Health and Pathobiology, College of Veterinary Medicine, North Carolina State University, Raleigh, North Carolina, USA

**Keywords:** *Clostridium difficile*, bile acids, metabolome, microbiota, obesity, therapeutics

## Abstract

Over the past 10 years, microbiome research has focused on defining the structures associated with different disease states in multiple systems, but has fallen short on showing causation. Prior omic studies have generated many new hypotheses, but moving forward we need to start dissecting the function of each bacterium alone and in concert with complex bacterial communities in well-characterized systems. Over the next 5 years, we need a merging of new omic technologies for exploratory studies with classical bacterial genetics, bacterial physiology, protein engineering, and biochemistry to further define the biochemical mechanisms of the gut microbiota. The future of the systems microbiology field will focus on targeted engineering and editing of the microbiome to alter function, which will be leveraged to prevent and/or treat human diseases. This perspective will focus on my contribution to the microbiome field, both past and present, and where I think research in the field is headed in the near future.

## Abstract (precis)

Over the past 10 years, microbiome research has focused on defining the structures associated with different disease states in multiple systems, but has fallen short on showing causation. Prior omic studies have generated many new hypotheses, but moving forward we need to start dissecting the function of each bacterium alone and in concert with complex bacterial communities in well-characterized systems.

## PAST AND PRESENT: FROM STRUCTURE TO FUNCTION OF THE GUT MICROBIOTA IN *C. DIFFICILE* INFECTION

My research has spanned the fields of protein biochemistry, molecular microbiology, microbial ecology, metabolomics, bacterial physiology, infectious disease, and pathogenesis. This multidisciplinary training has fostered an ability to think outside the box when solving real world problems. During my graduate career at North Carolina State University, I worked with advisor Amy Grunden to engineer and optimize archaeal proteins from *Pyrococcus* species for stable and long-term detoxification of nerve agents (1). To build upon my prior research training and to contribute to public health research, I completed my postdoctoral training with Vincent Young at the University of Michigan Medical School. My postdoctoral research training focused on exploring the interplay between the gastrointestinal tract (GIT) microbiota, and metabolome and the pathogen *Clostridium difficile*, a significant and reemerging public health problem. My research has shown that antibiotics disrupt the indigenous gut microbiota and, more importantly, the metabolome, reducing resistance to *C. difficile* colonization. My body of research on *C. difficile* has shifted the paradigm from focusing on the structure of the gut microbiota to the function, which is the metabolome (2). Since then, I have worked to marry the two concepts of engineering bacterial proteins for optimal specificity and activity and manipulation of the gut microbiota in health and disease. We are finally at a point in the microbiome field where we can bridge these two concepts, which will aid in the development of novel targeted therapeutics to treat infectious diseases.

The bacterial pathogen *C. difficile* is exquisitely sensitive to changes in the gut microbiota and metabolome, making it an attractive bacterium to study when designing new bacterial targets to manipulate the gut microbiota to restore colonization resistance. *C. difficile* is an anaerobic spore-forming Gram-positive bacillus that is the causative agent of *C. difficile* infection (CDI). There are an estimated 500,000 cases of *C. difficile* reported in the United States each year and an associated 29,000 deaths (3). Antibiotic exposure is a major risk factor for CDI; however, newer community-acquired cases with no prior exposure to antibiotics or the hospital are changing the epidemiology of this disease, as reviewed in reference 4. Even though antibiotics are a risk factor for CDI, they continue to be the first line of defense against this infection. After successful treatment, there continues to be a high rate of relapse (around 20 to 30%), making new therapies urgently needed (4). Over the past 7 years, we have used a combination of *in vitro*, *ex vivo*, and *in vivo* models to study the gut microbiome in the context of *C. difficile* colonization and disease. The goal was first to understand what *C. difficile* requires for growth and disease *in vitro* and then move to our *in vivo* models. We are now using this information against *C. difficile* to design novel therapeutics to restore colonization resistance against *C. difficile* in the gut. Work from my past and present research, and the work of others, has shown that antibiotics alter the structure of the gut microbiota, and this is associated with susceptibility to *C. difficile* colonization (5). In order to move beyond structure, we applied metabolomics to define what *C. difficile* was coming into contact with in the gut lumen. Metabolomics is a powerful tool that leverages mass spectrometry technologies to define the small molecules or metabolites within a system—in this case the GIT. There are other omic technologies that are able to help define the function of a system, including metagenomics, metatranscriptomics, and metaproteomics, but I would argue that metabolomics is the closest to function and the actual mechanism (6). Our work suggests there are two mechanisms that contribute to colonization resistance against *C. difficile*: (i) the gut microbiota produces secondary bile acids that inhibit *C. difficile* growth, and (ii) members of the gut microbiota are able to outcompete *C. difficile* for required nutrients, specifically amino acids (5). We are currently exploring both of these mechanisms alone and in concert using a variety of different approaches. We are also fortunate to have robust tractable animal models of CDI, making testing new bacterial therapeutics a reality (7).

## CURRENT ADVANCES: ENGINEERED BUGS TO TREAT GI AND METABOLIC DISEASES

My research on the gut microbiome and metabolome in the context of CDI has led me down different research paths, including one that focuses on bile acids, which are both host- and microbiota-derived metabolites that are able to shape the gut microbiota and the host. The indigenous gut microbiota is important for human health. Alterations to this microbial community influence bile acid metabolism and are associated with the development of obesity, diabetes, inflammatory bowel disease, colon cancer, and other gastrointestinal (GI) diseases, including CDI, as already discussed. Bile acids are synthesized by the liver from cholesterol and are essential for lipoprotein, glucose, drug, and energy metabolism. Primary bile acids are made by the host and make their way through the small intestine, where 95% of bile acids are absorbed in the terminal ileum through the enterohepatic system. The remaining bile acids that reach the large intestine are further biotransformed by members of the gut microbiota via dehydroxylation into secondary bile acids. Primary and secondary bile acids can also be conjugated and deconjugated with amino acids (either glycine or taurine) throughout the GIT, further feeding the gut microbiota. Bile acids can also act as detergents and further alter the microbiota and the host physiology, as reviewed in references 8 and 9. Therefore, we can use bile acids and the bacteria that are able to alter them to manipulate the gut microbiota and host physiology during different disease states (10).

Members of the gut microbiota encode proteins that are able to alter the bile acid pool in the gut. My group is interested in two enzymatic reactions: deconjugation of conjugated bile acids by bile salt hydrolases (BSHs [*bsh*]) and dehydroxylation of primary to secondary bile acids by the bile acid-inducible (*bai*) operon. Even though bacterium-derived bile acids play an essential role in host metabolism, little is known about their regulation or the bacterial enzymes able to modify them. Biochemical characterization studies for the bacteria that encode these proteins and the enzymes alone are lacking, allowing our team to fill this much-needed gap. We have designed a novel platform to identify, engineer, optimize, and biochemically characterize new BSHs capable of altering conjugated bile acids and to define their ability to influence the bile acid pool, gut microbiota, and host physiology *in vivo*. In order to do this, we also need a robust organism for delivery and expression *in vivo* and have selected *Lactobacillus* as our model system (11). This work will result in the construction of genetically modified *Lactobacillus* strains for therapeutic interventions to rationally alter the bile acid pool in the gut, modulating the gut microbiota and host physiology in GI and metabolic diseases. The novelty of this approach can be applied to optimizing other gut microbial enzymes that are critical to human health, including those important for short-chain fatty acid (SCFA) metabolism, specifically butyrate production, and secondary bile acid production. This information is expected to lead to unique therapeutic approaches for the prevention and treatment of GI and metabolic diseases.

## FUTURE CONSIDERATIONS: BIOTECHNOLOGICAL APPLICATIONS OF MICROBIOME-ENCODED ENZYMES

The future of the systems microbiology field is going to be focused on targeted engineering and editing of the microbiome to alter function, which will be leveraged to prevent and/or treat human diseases. We have spent a lot of time and money defining the structure of the microbiome associated with different disease states in multiple systems, but we have fallen short on showing causation. Prior omic studies have generated many new hypotheses, but moving forward we need to start dissecting the function of each bacterium alone and in concert with complex bacterial communities in each system (12). The biochemical activity of the gut microbiome still remains a large gap in our knowledge (13, 14). Over the next 5 years, we will need a merging of new omic technologies for exploratory studies with classical bacterial genetics, bacterial physiology, protein engineering, and biochemical characterization to further define the function of the microbiome. The advent of newer model systems that range in complexity to test these hypotheses, such as organoids and bioreactors, will be advantageous in dissecting microbiome function. Newer therapeutics precisely targeting the microbiome, like engineered bacteriophages and small molecules, will be important, as well as genetically tractable organisms for delivery in well-characterized systems (15).
